# Socioeconomic Factors and Their Role in Metabolic Dysfunction‐Associated Steatotic Liver Disease: A Comprehensive Review

**DOI:** 10.1111/liv.70731

**Published:** 2026-06-12

**Authors:** Leke Wiering, Münevver Demir

**Affiliations:** ^1^ Department of Hepatology and Gastroenterology, Campus Virchow‐Klinikum and Campus Charite Mitte, Corporate Member of Freie Universitat Berlin and Humboldt‐Universitat Zu Berlin Charite – Universitatsmedizin Berlin Berlin Germany

## Abstract

Metabolic dysfunction‐associated steatotic liver disease (MASLD), a major public health concern, is influenced by an interplay of genetic, environmental, and lifestyle factors, with socioeconomic factors functioning as important upstream determinants whose roles remain not yet fully understood. This review synthesizes current evidence on how different indicators of socioeconomic status as well as related social determinants of health—income, poverty, food insecurity, health insurance, education, migration, and composite indices—relate to MASLD prevalence, severity, and outcomes across different world regions. Several studies associate these socioeconomic factors with an increased risk of MASLD, illustrating the disease's link with socioeconomic disadvantages. However, socioeconomic factors often lose or attenuate their independent association with MASLD once key downstream determinants—such as diet quality, physical inactivity, and metabolic comorbidities—are considered, supporting a model in which socioeconomic factors mainly shape exposure to metabolic and behavioural risk factors rather than exerting a direct causal effect. Among the factors examined, education and food insecurity demonstrate the most consistent independent associations with MASLD. Notably, the direction of socioeconomic gradients appears to differ by regional income level, with lower SES associated with higher MASLD burden in high‐income countries but an inverse pattern in several middle‐income settings. Evidence remains largely limited to the United States, Europe, and a small number of Asian cohorts, underscoring the need for more geographically diverse research. This review highlights the association of different socioeconomic factors with MASLD, while also revealing the need for more detailed studies which systematically disentangle individual and area‐level social determinants of health, model mediating pathways and incorporate underrepresented regions and paediatric populations. A deeper understanding of how socioeconomic factors and downstream mediators jointly drive MASLD could inform targeted clinical strategies and multi‐level policies aimed at mitigating the social gradient in MASLD.

## Key Points

- Socioeconomic factors are associated with metabolic dysfunction‐associated steatotic liver disease (MASLD) prevalence, severity, and outcomes, but the strength and direction of association vary by indicator and setting.
- Food insecurity shows one of the most consistent independent associations with MASLD, while the association of income often attenuates after adjustment for downstream behavioural and metabolic factors.
- Higher educational attainment is generally associated with lower risk of advanced liver disease, particularly in US and European cohorts.
- Geographic variability: In high‐income settings, lower income and food insecurity generally correlate with greater MASLD burden, whereas an inverse gradient is observed in several middle‐income settings.
- Need for Further Research: The interrelationships and mediating pathways between different socioeconomic factors remain poorly understood. More comprehensive, geographically diverse studies with clearly defined socioeconomic factors are needed to inform screening strategies and multi‐level interventions.

**Abbreviations**
BMI: body mass index
GBD: global burden of disease study
MASH: metabolic dysfunction‐associated steatohepatitis
MASLD: metabolic dysfunction‐associated steatotic liver disease
MetALD: MASLD and increased alcohol intake
NAFLD: non‐alcoholic fatty liver disease
NHANES: national health and nutrition examination survey
SDI: socio‐demographic index
SES: socioeconomic status

## 1 Introduction

Metabolic dysfunction‐associated steatotic liver disease (MASLD) represents a disease of both medical and social relevance. With its prevalence rising in recent years [1] MASLD (formerly known as non‐alcoholic fatty liver disease, NAFLD) [2] has emerged as a global health concern [3] closely intertwined with the increase of obesity and metabolic disorders [4]. On an individual level, various well‐established risk factors for the development and progression of MASLD have been described. These include unfavourable dietary patterns rich in fructose and saturated fatty acids—leading to insulin resistance and other aspects of the metabolic syndrome [5, 6]. Additionally, genetic predispositions (e.g., PNPLA3) and effects of the microbiome have been identified [7, 8]. Beyond individual‐level determinants, socioeconomic factors have been recognized as central determinants of the incidence and outcomes of various diseases including diseases defining MASLD, such as diabetes and metabolic syndrome [9, 10]. This comprehensive review aims to explore the interaction between socioeconomic factors and MASLD [2]. We use the term ‘socioeconomic factors’ to encompass indicators of socioeconomic status (SES) (e.g., income), related social determinants of health (e.g., food insecurity) as well as composite measures of these factors. Beyond solely documenting social gradients in MASLD, an important methodological challenge is distinguishing between SES as a proxy for upstream social conditions and the downstream metabolic and behavioural factors that more directly drive liver fat accumulation and fibrosis. In various epidemiological studies the apparent effect of socioeconomic factors on MASLD depends strongly on the covariates included in statistical models: Once factors including adiposity, diabetes, diet quality, or physical activity are accounted for, indicators of SES and social determinants of health often lose or attenuate their independent association. In this review we therefore aim to interpret these findings with particular attention to adjustment sets and—where available—explicitly reported mediators and highlight how differences in these modelling strategies may explain heterogeneous results across populations and world regions.

## 2 Socioeconomic Factors and Their Associations With MASLD

**FIGURE 1:**
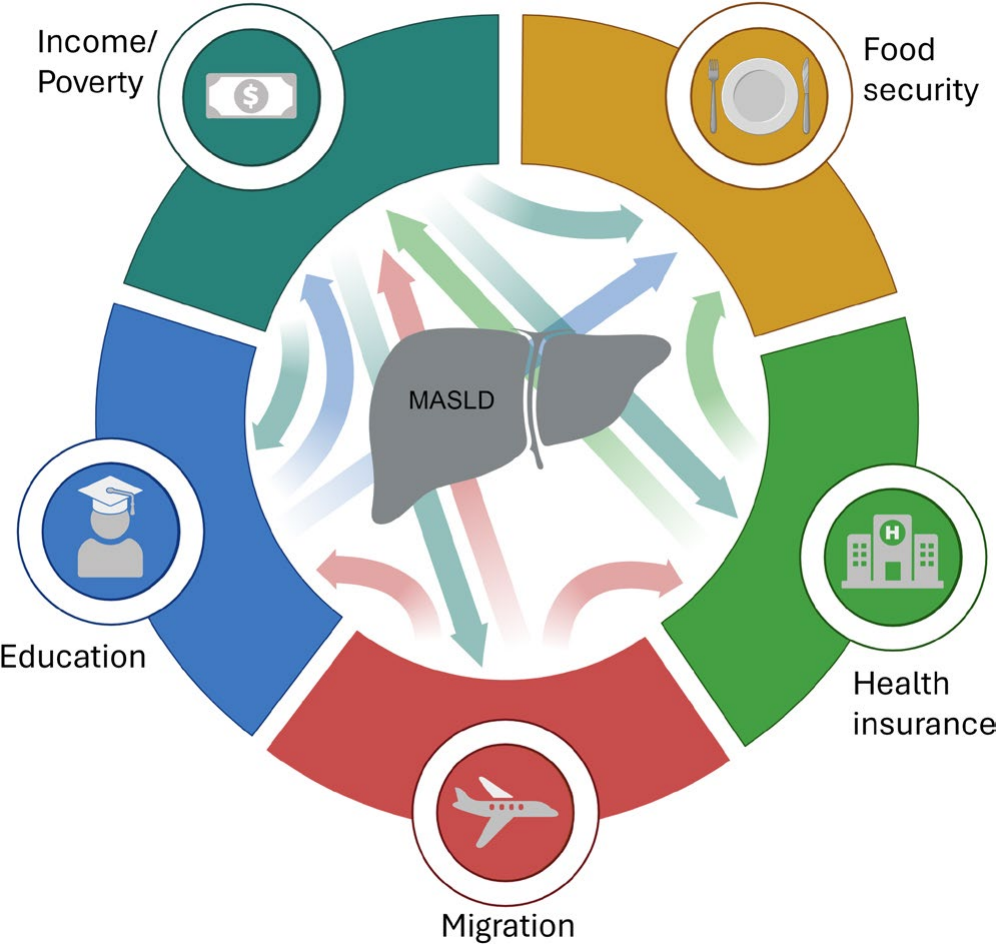
Social determinants of health influencing MASLD.

Understanding the diverse socioeconomic factors is pivotal for interpreting their relationship with MASLD. In this section we summarize how socioeconomic factors, including two core indicators of SES (income and education) as well as additional social determinants of health (food insecurity, health insurance status, and migration) and composite measures of SES, relate to MASLD prevalence, severity, and outcomes (Figure 1, Tables 1, 2, 3, 4, 5, 6, 7). Where possible, we distinguish between unadjusted and adjusted results and highlight factors mediating the association with MASLD.

**TABLE 1:** Studies analysing the impact of income on MASLD.

	Country	Study period	Study design	Population	Assessment of income	Data source of socioeconomic data	Primary endpoint	Definition of MASLD	Statistical analysis of socioeconomic data
Vilar‐Gomez et al., 2022 [11]	US	2017–2018	Cross‐sectional study	*n* = 3589 adults ≥ 18 years with negative serology for HBV and HCV without significant alcohol intake	Self‐reported using family poverty income ratio (PIR)	National Health and Nutrition Examination Survey (NHANES) [waves 2017–2018]	Prevalence NAFLD without fibrosis prevalence NAFLD with fibrosis	NAFLD: CAP ≥ 285 dB/m in VCTE Significant fibrosis: ≥ 8.6 kPa in VCTE	Ordinal logistic regression adjusted for: age, sex, ethnicity, BMI, waist circumference, education, physical activity, energy intake, diet quality
Golovaty et al., 2020 [12]	US	2005–2014	Cross‐sectional study	*n* = 2627 low‐income adults (≤ 200% of the federal poverty level) without chronic viral hepatitis or self‐reported heavy alcohol use	Self‐reported	National Health and Nutrition Examination Survey (NHANES) [waves 2005–2014]	Prevalence NAFLD prevalence fibrosis	NAFLD: US Fatty Liver Index (ultrasound, race/ethnicity, age, waist circumference, GGT, fasting insulin, fasting glucose) Fibrosis: NAFLD fibrosis score (age, BMI, diabetes/impaired fasting glucose, AST, ALT, platelets, albumin)	Logistic regression adjusted for: age, sex, ethnicity, household size, education, smoking, alcohol intake
Giammarino et al., 2022 [13]	US	2015–2020	Cross‐sectional study	*n* = 614 patients with histologically proven NAFLD/NASH without relevant alcohol consumption from a large tertiary health care network in New York	Not self‐reported but matched by zip code	American Community Survey from 2011 to 2015 by US Bureau of the census	Prevalence NASH prevalence significant steatosis prevalence significant fibrosis	NAFLD: ≥ 5% Steatosis and NAS ≥ 1 in liver biopsy NASH: NAS ≥ 4 in liver biopsy significant fibrosis: ≥ F2 severe steatosis: > 33%	No adjusted analysis for income
Ren et al., 2023 [14]	Netherlands	2010–2020	Nested case–control study (within prospective cohort)	*n* = 3152 adults aged 40–75 years	Self‐reported	The Maastricht Study	Prevalence NAFLD	NAFLD: hepatic stea‐tosis with intrahepatic lipid content ≥ 5.56% in MRI in the absence of excessive alcohol intake	Linear regression adjusted for: age, sex, diabetes, education, occupation; physical activity, dietary intake (fructose, fibre, vitamin E, alcohol, fat, protein)
Männistö et al., 2021 [15]	Finland	1992–2012	Prospective cohort study	*n* = 10 993 adults with NAFLD ≥ 25 without chronic viral hepatitis, without excessive alcohol consumption	Self‐reported via interviews and questionnaires	FINRISK Health 2000	Fatal and non‐fatal advanced liver disease requiring hospital admission or causing liver cancer, or liver‐related death	NAFLD: fatty liver index (FLI) ≥ 60 (BMI, waist circumference, GGT and triglycerides)	Fine‐Grey competing‐risk regression adjusted for: age, sex, BMI, diabetes, number of metabolic risk factors, cohort, polygenic risk score (PRS‐5)
Hu et al., 2020 [16]	China	2015	Cross‐sectional study	*n* = 1277 adults without excessive alcohol consumption and without other causes of liver disease	Not stated	Individual study (sub‐cohort from the “Diabetes Prevention Program”)	Prevalence NAFLD	NAFLD: liver fat content (ultrasound)	Logistic regression adjusted for: age, sex, BMI, blood pressure, smoking, alcohol intake, sodium intake, HbA1C, TG, TC, LDL, HDL, ALT, AST, uric acid
Zhou et al., 2019 [17]	China	2013–2014	Cross‐sectional study	*n* = 316 adults aged 26–76 years without significant alcohol drinking or HBV or HCV infection	Self‐reported annual household income	Individual study	Prevalence NAFLD	NAFLD: ratio of houns‐field units liver to hounsfield units spleen ≤ 1.1 on unenhanced abdominal CT	Logistic regression adjusted for: age, sex BMI, blood pressure, HbA1c, TC, TG, ALT, uric acid, postmenopausal status education, physical activity level, smoking, red meat intake, alcohol consumption
Zhang et al., 2022 [18]	China	2013–2019	Prospective cohort study	*n* = 16 168 adults aged 18–90 years without liver disease at baseline	Self‐reported using questionnaires	Tianjin Chronic Low‐grade Systemic Inflammation and Health (TCLSIH) Cohort Study	Incidence NAFLD	NAFLD: sonographic fatty liver in the absence of significant alcohol intake	Cubic spline regression adjusted for: age, sex, BMI, smoking, alcohol consumption, education, occupation, physical activity, energy intake, family history, diet quality

Abbreviations: ALT, alanine aminotransferase; AST, aspartate aminotransferase; BMI, body mass index; CAP, controlled Attenuation Parameter; CT, computed tomography; GGT, γ‐glutamyltransferase; HBV, Hepatitis B; HCV, Hepatitis C; HDL, high‐density lipoprotein cholesterol; LDL, low‐density lipoprotein cholesterol; MASLD, metabolic dysfunction‐associated steatotic liver disease; MRI, Magnetic resonance Imaging; NAFLD, Non‐alcoholic fatty liver disease; NAS, NAFLD activity score; NASH, non‐alcoholic steatohepatitis; TC, total cholesterol; TG, triglycerides; VCTE, Vibration‐controlled transient elastography.

**TABLE 2:** Studies analysing the impact of food insecurity on MASLD.

	Country	Study period	Study design	Population	Assessment of food insecurity	Data source of socioeconomic data	Primary endpoint	Definition of MASLD	Statistical analysis of socioeconomic data
Golovaty et al., 2020 [12]	US	2005–2014	Cross‐sectional study	*n* = 2627 low‐income adults (≤ 200% of the federal poverty level) without chronic viral hepatitis or self‐reported heavy alcohol use	Self‐reported using US Department of Agriculture's Core Food Security Modul (18‐item questionnaire)	National Health and Nutrition Examination Survey (NHANES) [waves 2005–2014]	Prevalence NAFLD prevalence fibrosis	NAFLD: US Fatty Liver Index (ultrasound, race/ethnicity, age, waist circumference, GGT, fasting insulin, fasting glucose) Fibrosis: NAFLD fibrosis score (age, BMI, diabetes/impaired fasting glucose, AST, ALT, platelets, albumin)	Logistic regression adjusted for: age, sex, ethnicity, household size, income, education, smoking, alcohol intake
Kim et al., 2024 [19]	US	2017–2018	Cross‐sectional study	*n* = 3441 adults ≥ 20 years without significant alcohol consumption or HBV or HCV	Self‐reported using US Department of Agriculture's Core Food Security Modul (18‐item questionnaire)	National Health and Nutrition Examination Survey (NHANES) [waves 2017–2018]	Prevalence MASLD	MASLD: CAP ≥ 263 dB/m in VCTE and one or more cardiometabolic criteria significant fibrosis: ≥ 8.0 kPa in VCTE	Logistic regression adjusted for: age, sex, ethnicity, diabetes, obesity, hypertension, TC, economic status, household size, education, marital status, physical activity, calorie intake, smoking
Kardashian et al., 2022 [20]	US	1999–2014	Prospective cohort study (population‐based)	*n* = 4816 adults with NAFLD *n* = 1654 adults with NAFLD with (advanced fibrosis)	Self‐reported using US Department of Agriculture's Core Food Security Modul (18‐item questionnaire)	National Health and Nutrition Examination Survey (NHANES) [waves 1999–2014] National Death Index data	All‐cause mortality health care utilization	NAFLD: US Fatty Liver Index (including ultrasound) Advanced fibrosis: NAFLD fibrosis score (NFS), APRI, or Fib‐4	Cox and logistic regression adjusted for: age, sex, ethnicity, diabetes, obesity, hypertension, hyperlipidaemia, education, income, insurance, smoking, diet quality, alcohol use
Kardashian et al., 2022 [21]	US	2017–2018	Cross‐sectional study	*n* = 1351 adults ≥ 20 years with NAFLD	Self‐reported using US Department of Agriculture's Core Food Security Modul (18‐item questionnaire)	National Health and Nutrition Examination Survey (NHANES) [waves 2017–2018]	Prevalence food insecurity in NAFLD patients	NAFLD: CAP ≥ 280 dB/m in VCTE in the absence of viral hepatitis or heavy alcohol intake significant fibrosis: ≥ 8.0 kPa in VCTE	Linear and logistic regression adjusted for: age, sex, ethnicity, BMI, income, education, physical activity, caloric intake
Tutunchi et al., 2021 [22]	Iran	2019	Cross‐sectional study	*n* = 210 adults 20–50 years without viral hepatitis, acute or chronic liver failure, and liver transplantation, and history of alcohol consumption in the last year	Self‐reported using US Department of Agriculture's Core Food Security Modul (18‐item questionnaire)	Individual study	Prevalence NAFLD	NAFLD: ultrasound	Logistic regression adjusted for: BMI, body fat percentage, waist circumference, waist‐to‐hip ratio, waist‐to‐height ratio, depression, physical activity, education, income, economic status, family size, family status, employment
Younossi et al., 2024 [23]	World‐wide	2021	Population‐based observational study/ecological		Not self‐reported using varying data sources per country	Global Burden of Disease Study 2021 United Na‐tions' country‐level food security data	MASLD prevalence MASLD liver‐related mortality	MASLD: PubMed search	Mixed‐effects linear regression adjusted for: country‐level median age, percentage of males, SDI

Abbreviations: ALT, alanine aminotransferase; APRI, AST to platelet ratio index; AST, aspartate aminotransferase; BMI, body mass index; CAP, controlled attenuation parameter; GGT, γ‐glutamyltransferase; HBV, hepatitis B; HCV, hepatitis C; HIV, human immunodeficiency Virus; MASLD, metabolic dysfunction‐associated steatotic liver disease; MRI, magnetic resonance Imaging; MRE, magnetic resonance elastography; NAFLD, non‐alcoholic fatty liver disease; SDI, social deprivation index; TC, total cholesterol; VCTE, Vibration‐controlled transient elastography.

**TABLE 3:** Studies analysing the impact of health insurance on MASLD.

	Country	Study period	Study design	Population	Assessment of health insurance	Data source of socioeconomic data	Primary endpoint	Definition of MASLD	Statistical analysis of socioeconomic data
Giammarino et al., 2022 [13]	US	2015–2020	Cross‐sectional study	*n* = 614 patients with histologically proven NAFLD/NASH without relevant alcohol consumption from a large tertiary health care network in New York	Not self‐reported but matched by zip code	American Community Survey from 2011 to 2015 by US Bureau of the census	Prevalence NASH prevalence significant steatosis prevalence significant fibrosis	NAFLD: ≥ 5% Steatosis and NAS ≥ 1 in liver biopsy NASH: NAS ≥ 4 in liver biopsy significant fibrosis: ≥ F2 severe steatosis: > 33%	No adjusted analysis for health insurance
Adejumo et al., 2019 [24]	US	2007–2014	Retrospective cohort study	*n* = 210 660 hospitalized patients with NAFLD without other causes of liver disease	Individual data	Healthcare Cost and Utilization Project Nationwide Inpatient Sample (HCUP‐NIS)	In‐hospital mortality discharge disposition length of stay cost	NAFLD: ICD‐code	Logistic, negative binominal and gamma regression adjusted for: age, sex, ethnicity, income quartile (by ZIP code), hospital region, hospital teaching status, Charlson‐Deyo comorbidity index cirrhosis stage
Noureddin et al., 2020 [25]	US	Cohort entry 1993–1996; follow‐up > 20 years	Nested case–control study (within a prospective cohort)	*n* = 2974 NAFLD patients *n* = 29 474 controls both groups Medicare fee‐for‐service participants	Self‐reported	Multiethnic Cohort (MEC) Study	Prevalence NAFLD	NAFLD: Medicare claims and/or ICD codes for fatty liver without relevant alcohol consumption	No multivariable analysis on SES

Abbreviations: MASLD, metabolic dysfunction‐associated steatotic liver disease; NAFLD, non‐alcoholic fatty liver disease; NAS, NAFLD activity score; NASH, non‐alcoholic steatohepatitis; SES, socioeconomic status.

**TABLE 4:** Studies analysing the impact of education on MASLD.

	Country	Study period	Study design	Population	Assessment of education	Data source of socioeconomic data	Primary endpoint	Definition of MASLD	Statistical analysis of socioeconomic data
Vilar‐Gomez et al., 2022 [11]	US	2017–2018	Cross‐sectional study	*n* = 3589 adults ≥ 18 years with negative serology for HBV and HCV and without significant alcohol intake	Self‐reported educational attainment	National Health and Nutrition Examination Survey (NHANES) [waves 2017–2018]	Prevalence NAFLD without fibrosis prevalence NAFLD with fibrosis	NAFLD: CAP ≥ 285 dB/m in VCTE significant fibrosis ≥ 8.6 kPa in VCTE	Ordinal logistic regression adjusted for: age, sex, ethnicity, BMI, waist circumference, income, physical activity, energy intake, diet quality
Noureddin et al., 2020 [25]	US	Cohort entry 1993–1996; follow‐up > 20 years	Nested case–control study (within prospective cohort)	*n* = 2974 NAFLD patients *n* = 29 474 controls both groups Medicare fee‐for‐service participants	Self‐reported	Multiethnic Cohort (MEC) Study	Prevalence NAFLD	NAFLD: Medicare claims and/or ICD codes for fatty liver without relevant alcohol consumption	Conditional logistic regression adjusted for: BMI, cardio‐vascular disease, alcohol intake, coffee, soda, physical activity, energy intake, smoking
Rieman‐Klingler et al., 2024 [26]	US	2011–2017	Cross‐sectional study	*n* = 264 adults without other possible causes of SLD or other chronic liver disease and without decom‐pensated cirrhosis	Self‐reported using questionnaires	Data from three indivi‐dual studies (Twin and Family Study, Familial Cirrhosis Study, T2DM Study)	Prevalence advanced fibrosis	Advanced fibrosis: Magnetic Resonance Elastography (MRE) ≥ 3.63 kPa	Linear and logistic regression adjusted for: age, sex, BMI, ethnicity, PNPLA3 genotype, income, alcohol use
Le et al., 2020 [27]	US	2011–2016	Case–control study	*n* = 4538 adults without heavy drinking or viral hepatitis	Self‐reported educational attainment	National Health and Nutrition Examination Survey (NHANES) [waves 2011–2016]	Prevalence NAFLD	NAFLD: US Fatty Liver Index (ultrasound, race/ethnicity, age, waist circumference, GGT, fasting insulin, fasting glucose) Fibrosis: NAFLD fibrosis score (age, BMI, diabetes/impaired fasting glucose, AST, ALT, platelets, albumin)	Logistic and multi‐nominal logistic regression adjusted for: age, sex, ethnicity, metabolic syndrome, cardiovascular disease, foreign born, income, smoking
Ren et al., 2023 [14]	Netherlands	2010–2020	Nested case–control study (within prospective cohort)	*n* = 4001 adults aged 40–75 years	Self‐reported	The Maastricht study	Prevalence NAFLD	NAFLD: hepatic stea‐tosis with intrahepatic lipid content ≥ 5.56% in MRI in the absence of excessive alcohol intake	Linear regression adjusted for: age, sex, diabetes, income, occupation; physical activity, dietary intake (fructose, fibre, vitamin E, alcohol, fat, protein)
Nasr et al., 2024 [28]	Sweden	1987–2020	Case–control study (registry‐based)	*n* = 14 026 adults ≥ 18 years with MASLD without other liver diseases *n* = 128 034 controls	Individual data derived from registry	Swedish National Patient Registry	Major adverse liver outcomes	MASLD: ICD coding in registry MALO: cirrhosis, decom‐pensated cirrhosis, or HCC (ICD‐codes)	Logistic and Cox regression adjusted for: age, sex, cardio‐vascular disease, diabetes, extra‐hepatic cancer, depression, year of diagnosis
Koutny et al., 2023 [29]	Austria	2013–2020	Nested case–control study (within prospective cohort)	*n* = 8727 adults between 40 and 77 years without viral hepatitis and relevant alcohol consumption	Self‐reported educational attainment	Paracelsus 10.000 study	Prevalence NAFLD prevalence liver fibrosis	NAFLD: fatty liver index (FLI) (BMI, waist circumference, GGT and triglycerides) liver fibrosis: Fib‐4 score	Linear and logistic regression adjusted for: age, sex, BMI, diabetes, metabolic syndrome, income, marital status, employment
Männistö et al., 2021 [15]	Finland	1992–2012	Prospective cohort study	*n* = 10 993 adults with NAFLD ≥ 25 without chronic viral hepatitis, without excessive alcohol consumption	Self‐reported via interviews and questionnaires	FINRISK health 2000	Fatal and non‐fatal advanced liver disease requiring hospital admission or causing liver cancer, or liver‐related death	NAFLD: fatty liver index (FLI) ≥ 60 (BMI, waist circumference, GGT and triglycerides)	Fine‐Grey competing‐risk regression Multivariable regression adjusted for: age, sex, BMI, diabetes, number of metabolic risk factors, cohort, polygenic risk score (PRS‐5)

Abbreviations: BMI, body mass index; CAP, controlled attenuation parameter; GGT, γ‐glutamyltransferase; GWA, genome‐wide association; HBV, Hepatitis B; HCC, hepatocellular carcinoma; HCV, hepatitis C; MASLD, metabolic dysfunction‐associated steatotic liver disease; MRI, magnetic resonance Imaging; NAFLD, non‐alcoholic fatty liver disease; NAS, NAFLD activity score; NASH, non‐alcoholic steatohepatitis; VCTE, vibration‐controlled transient elastography.

**TABLE 5:** Studies analysing the impact of migration on MASLD.

	Country	Study period	Study design	Population	Assessment of migration	Data source of socioeconomic data	Primary endpoint	Definition of MASLD	Statistical analysis of socioeconomic data
Giammarino et al., 2022 [13]	US	2015–2020	Cross‐sectional study	*n* = 614 patients with histologically proven NAFLD/NASH without relevant alcohol consumption from a large tertiary health care network in New York	Not self‐ reported but matched by zip code	American Community Survey from 2011 to 2015 by US Bureau of the census	Prevalence NASH prevalence significant steatosis prevalence significant fibrosis	NAFLD: ≥ 5% Steatosis and NAS ≥ 1 in liver biopsy NASH: NAS ≥ 4 in liver biopsy significant fibrosis: ≥ F2 severe steatosis: > 33%	No adjusted analysis for migration
Nasr et al., 2024 [28]	Sweden	1987–2020	Case–control study (registry‐based)	*n* = 14 026 adults ≥ 18 years with MASLD without other liver diseases *n* = 128 034 controls	Individual data derived from registry	Swedish National Patient Registry	Major adverse liver outcomes ^60^	MASLD: ICD coding in registry MALO: cirrhosis, decom‐pensated cirrhosis, or HCC (ICD‐codes)	Logistic and Cox regression adjusted for: age, sex, diabetes, cardio‐vascular disease, extra‐hepatic cancer, depression, year of diagnosis
Rivera‐Esteban et al., 2023 [30]	Spain	2021–2022	Cross‐sectional study	*n* = 4338 adult inmates with at least one meta‐ bolic disorder, without concomitant liver diseases or alcohol risk consumption	Self‐reported	Individual study	Prevalence MASLD prevalence significant fibrosis	MASLD: CAP ≥ 275 dB/m in VCTE and one or more cardiometabolic criteria Significant fibrosis: ≥ 8.0 kPa in VCTE	No adjusted analysis for SES

Abbreviations: ALT, alanine transaminase; HCC, hepatocellular carcinoma; MASLD, metabolic dysfunction‐associated steatotic liver disease; NAFLD, non‐alcoholic fatty liver disease; NAS, NAFLD activity score; NASH, non‐alcoholic steatohepatitis; VCTE, vibration‐controlled transient elastography.

**TABLE 6:** Studies analysing the impact of a combination of socioeconomic factors on MASLD.

	Country	Study period	Study design	Population	Assessment of socioeconomic data	Data source of socioeconomic data	Primary endpoint	Definition of MASLD	Statistical analysis of socioeconomic data
Giammarino et al., 2022 [13]	US	2015–2020	Cross‐sectional study	*n* = 614 patients with histologically proven NAFLD/NASH without relevant alcohol consumption from a large tertiary health care network in New York	Not self‐reported but matched by zip code: social depri‐vation index (SDI) and education level health care, foreign‐born population	American Community Survey from 2011 to 2015 by US Bureau of the census	Prevalence NASH prevalence significant steatosis prevalence significant fibrosis	NAFLD: ≥ 5% Steatosis and NAS ≥ 1 in liver biopsy NASH: NAS ≥ 4 in liver biopsy significant fibrosis: ≥ F2 severe steatosis: > 33%	Logistic regression adjusted for: age, sex, ethnicity, BMI, diabetes
Chen et al., 2023 [31]	US	2010–2020	Retrospective cohort study	*n* = 15 904 patients with MASLD without baseline malig‐nancy other than non‐melanoma skin cancer	Not self‐reported but matched by census tract geographic level (occupation, income, edu‐cation, single parents)	Neighbour‐hood data: National Neighbour‐hood Data Archive	Overall mortality liver‐related events	MASLD: hepatic steatosis on imaging plus cardiometabolic risk factor	Cox and Fine‐Grey competing risk regression adjusted for: age, sex, ethnicity, BMI, diabetes, hypertension, dyslipidaemia
Cho et al., 2021 [32]	Korea	2014–2018	Cross‐sectional study	*n* = 5272 age ≥ 50 years without other causes of liver disease	Self‐reported using questionnaire on income and/or education	Korea National Health and Nutrition Examination Surveys (KNHANES)	Prevalence NAFLD	NAFLD: hepatic steatosis index (HSI) and the comprehensive NAFLD score (CNS)	Logistic regression adjusted for: age, hypertension, waist circumference, marital status, region, type of housing, smoking, alcohol consumption, regular exercise, menopause
Singh et al., 2015 [33]	India	2012–2013	Case–control study (retrospective)	*n* = 464 adults with NAFLD *n* = 181controls both groups without relevant alcohol consump‐tion and without other known liver disease	Self‐reported using questionnaire	Individual study	Prevalence NAFLD	NAFLD: ultrasonography	No adjusted analysis for SES
Sadeghian‐pour et al., 2023 [34]	Iran	2016–2018	Cross‐sectional study	*n* = 10 009 adults aged 35–70 years without chronic other liver diseases and alcohol consumption	Wealth index: self‐reported townsend deprivation index: not self‐reported but matched by zip code	Hoveyzeh cohort study	Prevalence NAFLD	NAFLD: fatty liver index (FLI) ≥ 60 (BMI, waist circumference, GGT and triglycerides)	Logistic regression adjusted for: age, sex, dyslipidaemia, residence type, smoking, physical activity, energy intake, debate, educational, skill level
Ge et al., 2020 [35]	World‐wide	1990–2017	Population‐based observational study/ecological	Population‐based observational study	Not self‐reported using varying data sources per country	Global burden of disease study 2017	Prevalence NAFLD	NAFLD: PubMed search	No adjusted regression analysis performed; but results were stratified by age, sex, SDI
Chen et al., 2022 [36]	World‐wide	1990–2019	Population‐based observational study/ecological	Population‐based observational study	Not self‐reported using varying data sources per country	Global Burden of Disease Study 2019	NAFLD incidence rate NAFLD prevalence rate NAFLD death rate NAFLD DALYs	NAFLD: PubMed search	No adjusted regression analysis performed; but results were stratified by age, sex, SDI
Zhang et al., 2021 [37]	World‐wide	1990–2017	Population‐based observational study/ecological	Population‐based observational study	Not self‐reportedusing varying data sources per country	Global burden of disease study 2017	DALYs of liver cancer due to NASH	NAFLD: PubMed search	No adjusted regression analysis performed; but results were stratified by age, sex, SDI

Abbreviations: DALYs, disability‐adjusted life years; MASLD, metabolic dysfunction‐associated steatotic liver disease; NAFLD, non‐alcoholic fatty liver disease; NAS, NAFLD activity score; NASH, non‐alcoholic steatohepatitis, SDI, social deprivation index.

**TABLE 7:** Studies analysing the impact of socioeconomic factors on MASLD in children.

	Country	Study period	Study design	Population	Assessment of socioeconomic data	Data source of socioeconomic data	Primary endpoint	Definition of MASLD	Statistical analysis of socioeconomic data
Tang et al., 2023 [38]	US	2017–2022	Cross‐sectional study	*n* = 1574 adolescent < 20 years without HBV or HCV	Parental income self‐reported using family poverty income ratio (PIR)	National Health and Nutrition Examination Survey (NHANES) [waves 2017–2020]	Hepatic steatosis	CAP in VCTE (limitation: the definition used in this study corresponds to SLD not MASLD specifically, though both largely overlap in children)	Linear regression adjusted for: age, sex, ethnicity, BMI, diabetes, waist circumference, physical activity, TG, HDL‐C, LDL‐C
Paik et al., 2024 [39]	US	2017–2018	Cross‐sectional study	*n* = 771 adolescents aged 12–18 year without other possible causes of liver disease	Parental income and education, food insecurity self‐reported using US Department of Agriculture Child Food Security Survey Module (9‐item questionnaire)	National Health and Nutrition Examination Survey (NHANES) [waves 2017–2018]	Prevalence MASLD prevalence significant fibrosis prevalence advanced fibrosis	MASLD: CAP ≥ 285 dB/m in VCTE and one or more cardiometabolic risk factor significant fibrosis: ≥ 8.0 kPa in VCTE Advanced fibrosis: ≥ 13. kPa in VCTE	Logistic regression adjusted for: age, sex, ethnicity, BMI, diabetes, hypertension, obesity, hyperlipidaemia, prediabetes, insulin resistance, high CRP‐level
Orkin et al., 2020 [40]	US	2009–2018	Cross‐sectional study	*n* = 579 age < 21 years with NAFLD	Community deprivation index (CDI) not self‐reported but matched by census tract:	American Community Survey from 2011 to 2015 by US Census Bureau	Onset NAFLD and severity	NAFLD: MRI‐based evidence of steatosis in the context of a negative workup for other liver diseases or biopsy‐confirmed NAFLD	No adjusted analysis for SES
Orkin et al., 2024 [41]	US	2017–2021	Cross‐sectional study	*n* = 73 patients < 21 years with NAFLD without concurrent liver disease	Food insecurity self‐reported using US Department of Agriculture's Core Food Security Modul (18‐item questionnaire)	Individual study	Prevalence food insecurity in NAFLD patients	NAFLD: biopsy proven	No adjusted analysis for SES
Maxwell et al., 2024 [42]	US	2006–2013	Prospective cohort study	*n* = 132 children from Latina mothers with a non‐high‐risk pregnancy	Food insecurity self‐reported by parents at child's age 4 using US Department of Agriculture's Core Food Security Modul (18‐item questionnaire)	Individual study	Prevalence MASLD	MASLD: ALT ≥ 95th % for age/gender plus BMI ≥ 85% at time of ALT measurement	Logistic regression adjusted for: age, sex, BMI
Kivimäkie et al., 2018 [43]	Finland	1980–2012	Prospective cohort study (population‐based)	*n* = 3002 age 3–18 years at baseline	Parental education, household income and unemploymen: self‐reported education, living situ‐ation, unem‐ployment: matched by neighbourhood (250m^2^ grid):	Cardiovascular Risk in Young Finns Study statistics Finland's grid database	Prevalence fatty liver	Fatty liver: ultrasound (limitation: the definition used in this study corresponds to SLD not MASLD specifically, though both largely overlap in children)	Logistic regression adjusted for: age, sex, place of birth, cumulative individual socioeconomic disadvantage
Laitinen et al., 2020 [44]	Finland	1980–2011	Prospective cohort study	*n* = 2042 age 3 – 18 years at baseline	Parental education, household income and unemploymen: self‐reported Education, living situ‐ation, unem‐ployment: matched by neighbourhood (250m^2^ grid):	Cardiovascular Risk in Young Finns Study Statistics Finland's grid database	Prevalence fatty liver	Fatty liver: ultrasound (limitation: the definition used in this study corresponds to SLD not MASLD specifically, though both largely overlap in children)	Logistic regression adjusted for: age, sex, childhood BMI, insulin, birth weight, place of birth
Hagström et al., 2021 [45]	Sweden	1992–2016	Case–control study (Nationwide population‐based)	*n* = 165 individuals ≤ 25 years with biopsy‐proven NAFLD *n* = 717 controls	Parental education and migration individual data derived from national registries	ESPRESSO cohort study	Prevalence NAFLD	NAFLD: biopsy proven	Conditional logistic regression adjusted for: age, sex, maternal BMI, maternal smoking, maternal education, country of birth, pre‐eclampsia, gestational diabetes
Ayonrinde et al., 2017 [46]	Australia	1989–2009	Prospective cohort study	*n* = 1170 adolescents aged 17 years at follow‐up	Parental income and education self‐reported	The Raine Study	Prevalence NAFLD	NAFLD: fatty liver score ≥ 2 in ultrasound (parameters: liver echotexture, deep attenuation, vessel blurring) and no extensive alcohol consumption	No adjusted analysis for SES
León‐Plasencia et al., 2021 [47]	Mexico	2017	Cross‐sectional study	*n* = 33 obese children aged 6–16 years	Parental income and education self‐reported using questionnaires	Individual study	Prevalence NAFLD	NAFLD: ultrasound	No adjusted analysis for SES

Abbreviations: ALT, alanine aminotransferase; BMI, body mass index; CAP, controlled attenuation parameter; CRP, C‐reactive protein; HBV, hepatitis B; HCV, hepatitis C; HDL‐C, high‐density lipoprotein cholesterol; LDL‐C, low‐density lipoprotein cholesterol; MASLD, metabolic dysfunction‐associated steatotic liver disease; MRI, magnetic resonance Imaging; NAFLD, non‐alcoholic fatty liver disease; SES, socioeconomic status; SLD, steatotic liver disease; TG, triglycerides; VCTE, vibration‐controlled transient elastography.

### 2.1 Income/Poverty

Income and poverty are core indicators of SES, and their associations with MASLD prevalence and severity have been investigated in studies from the United States, Europe, and China (Table 1).

#### 2.1.1 United States

Across US studies—largely derived from the National Health and Nutrition Examination Survey (NHANES)—lower income is consistently associated with MASLD in unadjusted analyses but does not emerge as an independent risk factor after accounting for downstream behavioural and metabolic variables [11, 12]. Although overall prevalence of MASLD as well as MASLD with advanced liver disease are both more common among individuals with lower income, these associations are attenuated in multivariable models. Instead, college education—partly mediated through higher diet quality and greater physical activity—as well as food insecurity were identified as independent risk factors. One biopsy‐based cross‐sectional study from New York did not confirm an association between lower income and higher rates of metabolic dysfunction‐associated steatohepatitis (MASH) or severe steatosis, though its reliance on neighbourhood‐level socioeconomic data rather than individual data and absence of adjustment for other risk factors limit its interpretability [13].

#### 2.1.2 Europe

European studies also point to an indirect role of income in MASLD, rather than a direct effect, operating through lifestyle and other socioeconomic mediators, similar to evidence from the US [14, 15]. In a Dutch cohort, lower household income was associated with a higher risk of MASLD, with effects partially mediated by physical activity and fructose intake [14]. A prospective Finnish MASLD cohort further showed that unemployment—an indicator of economic disadvantage—was associated with increased risks of both liver‐related outcomes as well as non‐liver‐related mortality, independent of metabolic comorbidities, though the study did not explore potential mediating pathways [15].

#### 2.1.3 Asia

In contrast to findings from high‐income countries, three Chinese studies suggest that higher income is associated with greater MASLD prevalence [16, 17, 18]. This association persisted after adjustment for metabolic comorbidities in two cross‐sectional studies, although underlying mechanisms were not examined. A large prospective cohort study provides a potential explanation: higher household income and higher educational attainment were associated with a greater consumption of ultra‐processed foods, which in turn independently predicted MASLD risk [18].

Overall, income rarely remained an independent determinant of MASLD once downstream behavioural and metabolic factors were considered. The direction of association appears to be region‐specific. In high‐income settings, lower income is linked to higher MASLD risk through adverse lifestyle patterns as well as co‐occurring metabolic comorbidities. By contrast, in middle‐income countries such as China, higher income may increase MASLD risk through greater consumption of energy‐dense, ultra‐processed foods, resulting in an association in the opposite direction to that observed in the United States and Europe.

### 2.2 Food Insecurity

Food insecurity, a distinct social determinant of health, and its association with MASLD have been examined in several studies, with most evidence originating from the United States (Table 2).

#### 2.2.1 United States

US studies consistently link food insecurity to MASLD prevalence, and in some analyses also to advanced fibrosis and adverse outcomes—an association that, where examined, persists after adjustment for income, education, caloric intake, and physical activity [12, 19, 20]. The association with fibrosis appears to be partly mediated by obesity and diabetes, whereas the association with MASLD prevalence persists after accounting for these factors [19]. Beyond disease prevalence, analysis of NHANES‐linked mortality records shows higher all‐cause mortality and greater healthcare utilisation among food‐insecure individuals with MASLD, again independent of education and income [20]. Dietary quality may partly explain these findings, although available evidence suggests only modest but heterogeneous differences in diet quality by food insecurity and ethnicity, with poorer diet quality most prominently observed among food‐insecure White patients [21].

#### 2.2.2 Asia

Outside the United States, evidence is limited to a single Iranian cross‐sectional study, which found food insecurity to be independently associated with higher prevalence and risk of MASLD, regardless of physical activity, education, or income [22]. This finding tentatively extends the US pattern to a middle‐income country, though generalisability is limited by the exclusion of patients with diabetes and hypertension.

#### 2.2.3 Global (Ecological)

At the global level, an ecological analysis of data from the Global Burden of Disease study (GBD) of 204 countries suggested that the association between food insecurity and MASLD differs by national income level: In high‐income countries, food insecurity was associated with a higher MASLD risk, whereas in low‐income settings it was associated with a lower risk [23]. This contrast may reflect differences between poor diet quality in high‐income settings and true undernutrition in low‐income settings.

Overall, food insecurity represents one of the most consistently documented social determinants of health associated with MASLD, with an independent association with disease presence, advanced fibrosis, and mortality demonstrated across multiple analyses. In high‐income countries, this association appears to be driven less by caloric excess and more by poor diet quality, obesity, and diabetes. In low‐ and middle‐income countries, where food insecurity may reflect true undernutrition, the relationship with MASLD appears more heterogeneous, underscoring the need for context‐specific studies that explicitly model mediating nutritional and metabolic factors.

### 2.3 Health Insurance

Health insurance, a distinct social determinant of health, has been examined in only a few studies in the context of MASLD (Table 3).

#### 2.3.1 United States

Available US studies link private or more stable insurance coverage primarily to better in‐hospital outcomes with limited evidence for an association with MASLD prevalence [13, 24, 25]. Evidence derives from a large retrospective cohort of over 200 000 hospitalised patients with MASLD, in which publicly insured or uninsured patients experienced higher mortality, longer hospital stays, and lower rates of discharge to home compared to patients with private health insurance, independent of patient and hospital characteristics [24]. Household income and comorbid burden were additional independent risk factors for these adverse outcomes. Beyond insurance type, continuity of coverage also appears relevant: A case–control study found that patients with advanced MASLD had shorter periods of continuous insurance coverage compared to those with MASLD without cirrhosis [25]. While this may suggest that gaps in coverage are relevant to disease progression, socioeconomic factors were not analysed as independent risk factors in this study, limiting direct conclusions.

Whether insurance status acts primarily as a proxy for higher SES and healthier lifestyles or exerts a more direct protective effect by facilitating earlier diagnosis, access to specialised care and use of emerging pharmacotherapies remains to be studied.

### 2.4 Education

Education, a core indicator of SES, is among the most frequently studied socioeconomic factors in relation to MASLD (Table 4).

#### 2.4.1 United States

Across US studies, lower educational attainment is associated with MASLD prevalence and—more consistently—advanced liver disease, with associations persisting after adjustment for metabolic risk factors as well as income, if multivariable statistical models were reported [11, 25, 26, 27]. Diet quality and physical activity appear to partly mediate this relationship [11]. Additional analyses suggest that education and other socioeconomic factors may attenuate the genetic risk of PNPLA3 and may also influence disease awareness, which is higher among more educated individuals [26, 27].

#### 2.4.2 Europe

European studies largely mirror findings from the United States, with higher educational attainment being associated with lower MASLD risk and more favourable liver outcomes [14, 28, 29]. These associations appear at least partly independent of income and employment and may be mediated by physical activity and diet quality. One Finnish cohort did not replicate this association, but co‐occurring socioeconomic factors were not jointly modelled, limiting interpretation [15].

In summary, higher educational attainment is consistently associated with a lower risk of advanced liver disease across US and European cohorts, even though its relationship with overall MASLD prevalence is more heterogeneous. Proposed mediators include physical activity, higher diet quality, and disease awareness, but formal mediation analyses are scarce. Notably, several studies report that the protective effect of education persists after adjustment for income, suggesting education captures advantages beyond income.

### 2.5 Migration

Migration is a social determinant of health that has not been well studied in relation to MASLD (Table 5).

#### 2.5.1 United States and Europe

The available evidence—drawn from the United States and Europe—is limited and inconsistent with no clear association between migration and MASLD [13, 28, 30]. A New York study reported higher odds of MASH and severe steatosis among migrants, but the analysis was unadjusted and relied on neighbourhood‐level socioeconomic data [13]. By contrast, Swedish and Spanish studies did not show a clear association with liver outcomes or MASLD prevalence [28, 30].

Overall, migration status appears to be an imprecise proxy for social disadvantage in MASLD. Existing studies do not account for language proficiency, legal status, healthcare access, occupation, or discrimination and therefore may conflate vastly different migrant experiences, ranging from refugees to highly skilled expatriates. Observed associations are therefore difficult to interpret and likely reflect a complex interplay of different socioeconomic factors. Future studies should therefore prioritise more granular, structural variables over crude binary migration indicators.

### 2.6 Composite SES Measures and Global Geographic Patterns

Some studies did not analyse individual socioeconomic factors but instead composite measures or indices (Table 6).

#### 2.6.1 United States

US studies using such composite measures consistently indicate that overall socioeconomic disadvantage is independently associated with higher MASLD risk and worse outcomes [13, 31]. The cross‐sectional study from New York found that the cumulative number of socioeconomic risk factors predicted MASH, with a trend emerging at three or more factors and a significant association at four or more risk factors, independent of demographic variables [13]. A large retrospective cohort study similarly found that composite affluence was associated with reduced overall mortality and liver‐related events, even after adjustment for patients' demographics and metabolic comorbidities [31]. Notably, in both studies, socioeconomic data was assigned geographically rather than self‐reported, which may introduce misclassification at the individual level.

#### 2.6.2 Asia

Findings from Asian studies are more heterogeneous and appear to vary by national economic context, consistent with the income‐specific patterns described in Section 1. In South Korea—a high‐income country—lower SES was associated with higher MASLD prevalence, mirroring the US pattern [32]. By contrast, studies from India and Iran—both middle‐income countries—report a contrary trend, where higher socioeconomic class is associated with an increased risk of MASLD [33, 34]. However, these studies used different composite measures and had limited adjustment for behavioural confounders (e.g., physical activity), restricting comparability.

#### 2.6.3 Global (Ecological)

Ecological studies, such as those based on the GBD, complement individual‐level findings by capturing broader global variations. Utilising the Socio‐Demographic Index (SDI)—a composite of per capita income, education, and fertility rates—GBD data show that the relationship between socioeconomic development and MASLD burden at a national level is heterogeneous across world regions. While MASLD prevalence has risen globally since 1990, the most substantial increases have been recorded in high‐middle SDI countries, whereas the greatest absolute incidence and relative growth were observed in middle and low‐middle SDI countries [35, 36]. Notably, higher SDI is associated with lower disease severity in both MASLD and MASH‐related hepatocellular carcinoma [36, 37]. These ecological findings need nonetheless to be interpreted cautiously. GBD estimates rely on modelled data, particularly in regions with limited diagnostic capacity or incomplete mortality reporting. Consequently, MASLD‐related estimates may rather reflect overall liver‐related mortality, and interpretation of prevalences and incidences should be contextualised accordingly [48].

In summary, studies using composite SES measures in high‐income countries consistently associate lower SES with higher MASLD prevalence and worse outcomes, whereas studies from middle‐income countries again suggest the opposite trend, reinforcing the pattern observed in income‐specific analysis.

### 2.7 MASLD In Children

The increasing prevalence of MASLD is observed not only in adults but also in children and adolescents, and several studies have explored the role of socioeconomic factors in this context (Table 7). Paediatric data broadly mirror adult findings, with socioeconomic disadvantage being associated with a greater burden of MASLD in high‐income settings, whereas an inverse gradient is observed in middle‐income settings.

#### 2.7.1 United States

In the US, low household income and food insecurity consistently emerge as key risk factors for MASLD in children, with food insecurity mediating the pathway between low income and increased disease risk [38, 39, 40, 41, 42]. Children from deprived backgrounds are diagnosed at a younger age, though not with greater disease severity at presentation in descriptive analyses [40, 41]. Food insecurity independently associates with increased MASLD risk, even after adjustment for age, sex, and BMI, underscoring the disproportionate burden in vulnerable populations [42].

#### 2.7.2 Europe

European longitudinal data highlight a long‐term impact of early‐life socioeconomic disadvantage on MASLD risk. The Young Finns Study, a unique 30‐year prospective cohort from Finland, found that low childhood SES independently predicted MASLD in adulthood, after adjustment for demographic and early‐life metabolic factors [43, 44]. Mediating factors included BMI, physical activity, blood pressure, insulin levels, lipid profiles, and red meat consumption. Socioeconomic differences in diet emerged by age six, physical activity by age 12, and BMI by age 21, indicating that the socioeconomic gradient in MASLD risk is established well before adulthood. Data on migration from a Swedish cohort study carry the same caveats discussed in section 5 [45].

#### 2.7.3 Mexico and Australia

The high‐ versus middle‐income divergence observed in adult studies is also found in children. In Australia (a high‐income country), higher family income predicts lower MASLD risk in adolescence, consistent with US and European patterns [46]. Conversely, a small study of Mexican children (a middle‐income country) with obesity found higher MASLD prevalence among those with higher parental education and higher SES, confirming the pattern observed in other middle‐income countries for adults [47]. Neither study reported adjusted results.

Taken together, income and food insecurity emerge as more robust independent risk factors for paediatric MASLD than parental education, though formal mediation analyses remain rare. The Young Finns Study uniquely shows how early‐life socioeconomic disadvantage shapes diet, physical activity, obesity, and cardiometabolic risk trajectories from childhood onward, eventually manifesting as fatty liver disease in adulthood. Evidence from low‐ and middle‐income countries and from ethnically diverse paediatric populations remains scarce, representing a critical gap given the high burden of MASLD in these groups.

## 3 Discussion

Overall, the available literature supports a meaningful association between socioeconomic factors and MASLD, but the strength, independence, and even direction of that association vary by indicator, outcome definition, and geographic setting. Income, food insecurity, and education have the strongest evidence base; among these, food insecurity demonstrates the most consistent independent association, whereas the effect of income often attenuates after adjustment for downstream behavioural and metabolic factors. A recurring regional pattern emerges across the results: Lower SES is associated with greater MASLD burden in high‐income countries, but with a lower burden in several middle‐income settings, likely reflecting differences in nutrition transition and access to energy‐dense, ultra‐processed foods. Even among countries with similar income levels, the generalisability of findings might be limited. While most European countries, like the United States, are high‐income countries, the applicability of American data to European countries and vice versa remains uncertain. The nature of government social support (e.g., unemployment benefits or food assistance programmes) and other support structures may be vastly different. Most European countries have national health insurance systems, whereas the American health insurance system is a mix of public and private coverage potentially leaving patients with high out‐of‐pocket costs. These structural differences in social protection and healthcare coverage likely contribute to divergent health outcomes and costs between the United States and European countries, as illustrated by higher unemployment‐related mortality and markedly higher annual MASH‐related medical costs in the United States [49, 50]. It is therefore possible that some of the study results from the United States may have been less pronounced if stronger social support systems were in place. However, several European studies also report an association between socioeconomic factors and MASLD suggesting that while currently existing social security systems may attenuate some socioeconomic disparities, they do not eliminate them.

While some relationship between socioeconomic factors and MASLD has been shown in most studies, the current body of evidence has various limitations, and several questions remain unanswered. As noted, data from several regions with high prevalence of MASLD, such as the Middle East and several South American and African countries, are sparse or missing [36, 51]. As we discuss above there seem to be relevant differences in the relation of low SES with MASLD between high‐ and middle‐ or low‐income countries. Whether this relation differs further within low‐income countries remains speculative, as studies below a national level are missing from these regions. Further research is urgently needed to better understand the association of socioeconomic factors and MASLD in low‐ and middle‐income countries. In addition to these contextual differences, heterogeneity in reporting of confounders and modelling strategies likely contributes to the heterogeneous effects of socioeconomic factors. Across studies, the strength and even direction of the association of socioeconomic factors with MASLD depend strongly on the covariates taken into account and the modelled outcomes: While low income or unemployment are often associated with higher MASLD prevalence or advanced fibrosis in minimally adjusted models, these effects often weaken or disappear after adjustment for diet quality and physical activity as well as food insecurity; in contrast, educational gradients in advanced liver disease frequently persist after controlling for income, but they are partly attenuated when lifestyle and comorbidity burden are included. This pattern supports a model in which socioeconomic factors rarely act as a direct cause of MASLD but instead structure exposure to proximal risk factors—unhealthy diet, physical inactivity, central obesity, insulin resistance, diabetes, and suboptimal healthcare access—which drive steatosis and fibrosis. Methodologically socioeconomic factors can function as confounders, mediators, or colliders depending on the causal question. Future studies should therefore pre‐specify hypothesised pathways and apply suitable methods to separate direct from indirect effects on MASLD.

Another limitation lies in the observational nature of most studies on socioeconomic factors and MASLD. Even though many of the discussed studies are large prospective cohort studies, they should still be interpreted with caution, as they cannot establish causal relationships. Regarding data quality, most studies relied on self‐reported socioeconomic information. However, some studies used area‐level socioeconomic indicators (e.g., zip‐code matched) rather than individual‐level data [13, 31, 40]. This approach may represent a limitation, as it may reduce the accuracy and specificity of the socioeconomic data. As an example, Giammarino et al. found no association between poverty and MASLD, a finding that contrasts with much of the literature and may be partly attributable to the use of matched, aggregate‐level socioeconomic data [13]. The definitions of MASLD used in the studies exhibit considerable differences. Some studies include only patients with biopsy‐confirmed MASLD or MASH [13], while others rely on various imaging methods (e.g., ultrasound [44], MRI [40]). Yet others use different scores based on laboratory parameters and clinical data [12, 20]. The heterogeneity of these inclusion criteria may also explain differing results. Uniform criteria based on guidelines would be desirable to achieve better comparability and transferability of results between different regions, countries, and social systems. In 2023, the definition and nomenclature of steatotic liver diseases were changed therefore several of the studies included in this review are still based on the previous definition of NAFLD [2]. However, it has been consistently reported that the diagnoses of NAFLD and MASLD exhibit a very high concordance [39, 52, 53]. Therefore, we are confident that findings from studies on socioeconomic factors in NAFLD can be reliably transferable to MASLD, provided that a proper definition of NAFLD was applied. In addition to pure MASLD, the disease entity of MASLD and increased alcohol intake (MetALD) was newly defined. For this newly described entity there are no valid data on its association with socioeconomic factors yet and new studies are needed to differentiate the role of socioeconomic factors in these patients with both aspects of metabolic syndrome and relevant alcohol consumption. While this review concentrates on the association of socioeconomic factors with MASLD, an individual's risk for developing MASLD is influenced by many other factors, including sex and ethnicity. These aspects often interact with socioeconomic factors, adding complexity to the relationship. Their impact on MASLD has been thoroughly addressed elsewhere and is beyond the scope of the present review [54, 55].

## 4 Implications for Clinical Practice and Policy

Despite these limitations, the available evidence already has implications for hepatology practice and public health. Clinicians should recognise low SES, including low educational attainment, as well as food insecurity, as markers of increased MASLD risk and potentially delayed diagnosis, and consider proactive case‐finding strategies, tailored counselling, and linkage to social and nutritional support services in these groups. At a policy level, interventions that improve access to affordable, healthy food, promote safe opportunities for physical activity in deprived neighbourhoods and ensure equitable coverage of non‐invasive liver assessment and specialist referral are likely to yield disproportionate benefits for socioeconomically disadvantaged populations. Finally, integrating SES and food insecurity screening into routine MASLD care and clinical trials could help identify high‐risk patients, refine risk stratification tools, and inform resource allocation.

## 5 Conclusion

In conclusion, this review highlights that socioeconomic factors are strongly associated with MASLD prevalence and outcomes across diverse settings, but rarely act as isolated, independent determinants. Income, poverty, food insecurity, health insurance, education, migration, and composite SES measures all relate to MASLD, predominantly by shaping exposure to downstream metabolic and behavioural risk factors and possibly modifying access to timely diagnosis and specialist care. Global variations—including opposing social gradients between high‐ and middle‐income countries—underscore the need to interpret findings within their regional social, nutritional, and healthcare contexts rather than extrapolating results across settings. Future research should systematically disentangle individual and area‐level socioeconomic factors, apply robust causal and mediation approaches, and explore the role of socioeconomic factors in the newly defined entities of MASLD and MetALD. Ultimately, integrating socioeconomic factors into risk stratification, clinical pathways, and multi‐level policy interventions will be essential to mitigate the social gradient in MASLD and ensure that advances in diagnostics and therapy translate into equitable liver health gains.

## Funding

The authors have nothing to report.

## Conflicts of Interest

The authors declare no conflicts of interest.

## Data Availability

Data sharing not applicable to this article as no datasets were generated or analysed during the current study.
